# Academy of Stomatology

**Published:** 1899-05

**Authors:** 

**Affiliations:** Academy of Stomatology


### ACADEMY OF STOMATOLOGY.

THE regular monthly meeting of the Academy of Stomatology was held at the rooms of the Academy, 1728 Chestnut Street, on the evening of January 24, 1899, Professor S. H. Guilford, president *pro tem.*

A paper entitled "Systemic Remedies in Diseases of the Teeth" was read by Professor Leo Greenbaum.

(For Professor Greenbaum's paper, see page 289.)

### DISCUSSION.

*Dr. Truman.*—I think it is altogether wrong that dentists, as a rule, do not prescribe internally. I can commend the use of asafœtida. The other remedies and the methods advised in their administration are very satisfactory, especially in case of chloral. I can see no objection to its use after the manner recommended. We do have, unquestionably, a great many cases of acute pericementitis and apical abscess. When patients are suffering, something more has to be done than mere counter-irritation, and we must use internal agents. The coal-tar derivatives and bromides are also very beneficial in many cases.

Dentists generally have an idea that they have no right to prescribe. It is time such an idea be given up. They have as much right to prescribe for dental conditions as the medical man has, and should exercise that right and write prescriptions. I find it hard to convince my students that it is their duty to have prescriptions properly filled and administered. We have adhered too long to the idea of local irritation. We need the benefits of internal as well as external medication.

I indorse the essayist's opposition to capsicum plasters as counter-irritants, having discarded them long ago. There are agents better for this purpose than capsicum. He does not mention turpentine, and its derivative terabine, which is one of the best, always at hand, easily applied, and better than capsicum. A piece of sterilized cottonoid saturated with it may be applied to the gum.

*Dr. Register.*—I use turpentine as a local remedy in combination with iodine or aconite quite extensively. It is very peculiar in its influence, being a resolvent in some cases, and in others it acts in an opposite manner, aiding the process of suppuration. I write a prescription to be filled by the druggist and applied by the patient with a camel's-hair brush. As our essayist and Dr. Truman have said, we as dentists ought to cease to be looked upon as simply mechanics. We can do very much more for our patients by the utilization of internal medication, together with our antiseptics, in the treatment of teeth, the relief of pain, or the anticipation of cures, than by any mechanical treatment. The techniques taught by the colleges are overrated. I think students should be thoroughly grounded in the fundamental principles that govern practice, and the techniques will come to them just as they come to surgeons or specialists who pick them up in after-life. Unless one has that knowledge that comes from the comprehension of fundamental principles, he can never adapt himself to the changes in dental technique. That which he does take up he must largely unlearn.

We ought to feel ourselves specialists in medicine. In my own practice whenever I have a patient with an abscess I do not hesitate to use the hypodermic syringe and morphine sulphate to the extent of thoroughly placing the patient under its influence so that he is relieved. If the patient cannot be relieved in that way, then, of course, we have the radical cure of extraction, but I do not think that people should be allowed to endure torture day after day until nature brings relief. On one occasion for relief of pulpitis caused by pulp-nodules I had to administer hypodermically, in divided doses, three-eighths of a grain of morphine sulphate to produce relief. When my patients dread the thought of dental ministrations I treat them by internal medication, such as small doses of aconite, to prepare them for operations by lessening the nervous irritability. For cleansing teeth I use a germicidal agent that is made of iodoform, salol dissolved in chloroform, and alcohol, to which is added a small quantity of oil of cinnamon or oil of cloves. I apply this with a camel's-hair brush or bibulous paper to the dry teeth, with or without rubber dam, allow it to remain for a minute or two, and follow with a mixture of iodine and chloroform, equal parts, and allow that to dry. The effect is simply marvellous. I never knew what it was to have my patients have clean teeth until I used this preparation. Since Williams has shown us how bacteria really act, we do get the teeth clean by taking off the zoöglœa of bacteria.

*Dr. Hickman.*—I cannot see how a capsicum plaster applied to the gum would draw pus from the root of a tooth. I should think that a poultice would do the work better. It seems to me that we always apply a counter-irritant to prevent pus formation and a poultice to cause pointing of an abscess. I would like to be informed.

*Dr. Curry.*—I wish to inquire regarding the medico-legal aspect of prescribing remedies. The essayist did not speak of that. Suppose a dentist prescribed, for example, chloral, and, owing to a weak heart action, the patient dies. There being no physician in attendance, and the prescription plainly showing what the patient took as the last remedy, would the coroner's inquest exonerate the dentist, or would the dentist have to prove his right to prescribe? Where would the responsibility rest?

*Dr. Guilford* (replying to Dr. Hickman).—A counter-irritant is applied to relieve the congestion about the root apex. A roasted fig or raisin formerly was applied as a poultice to cause pointing.

(Replying to Dr. Curry.) The court has held that a dentist has a perfect right to use an anæsthetic, and in case of accident he would be no more responsible for it than would a medical man. A man should have average intelligence and education in the matter, possess average skill, and use average care. I think the law requires nothing more than that. I do not know that the question, as asked, has been regularly tested in court, but it has been put in almost the same way, and I think that the courts have decided that a dentist has as much right to prescribe a medicine to relieve diseases of the teeth as a physician has to treat the general system.

*Dr. Truman.*—You are right, Mr. Chairman. In Rehfuss's "Jurisprudence" he will find that the law simply demands ordinary skill, and that if he has the proper authority to perform any operation the law will stand by him, just as it will by the medical man, and both the physician and the dentist are liable to suit for malpractice. The diploma does not save either of them.

According to my idea a counter-irritant acts thus: All irritants act on the sensory nerves and thus increase the flow of blood to a particular part. If applied to the gum-tissue, it excites these nerves, producing congestion of the blood-vessels upon the surface and a lessening of the blood-supply at the focus of inflammation. Pus seeks an exit through the least resistant tissue, this being accomplished by increased emigration of leucocytes and breaking down of tissue, producing tension and resorption. This is, possibly, intensified by some quality in the constituent of the pus not recognized; otherwise it is difficult to understand the tortuous canals (fistulæ) common in the oral cavity.

*Dr. Jeffries.*—For the most part I prefer local treatment, and have used cataphoresis repeatedly on hypersensitive dentine and in the extraction of pulps without previous treatment. I had a great deal of satisfaction in a case of removal of pulp-nodules from a tooth. For hypersensitive dentine I do not know any better local treatment than the old-fashioned carbonate of potassium in glycerin (saturated solution). I have used that remedy both with and without the rubber dam with as much comfort as could possibly be obtained with any other remedy. It is applied on a small pellet of cotton. In some cases I seal a small portion in a cavity, renewing it every day or two. It is effective even when dentine is so excessively sensitive that the touch of an explorer causes excruciating agony. This is not new, but it is effective. There used to be a fear that this remedy would affect the pulp, but I have never known of such a case. I did have, several years ago, a case of death of the pulp from the use of the cataphoric current, but in that case the tooth contained a large gold filling and the pulp received the force of the current too strongly.

*Dr. Schamberg.*—I wish to express my sentiments in regard to constitutional remedies in the treatment of the teeth and adjacent parts. There are undoubtedly cases where a patient, while suffering from a dental disease, may be afflicted with nervous excitability to such an extent that local remedies do not have the desired effect. Very often bromides have been serviceable in allaying this general condition. I am quite fond of the use of aconite. I find that the nervous excitability causes an acceleration of the heartaction and there is increased inflammation of an irritated part. By using aconite as a sedative in one- or two-drop doses every four to six hours, one very often gives decided relief. I might enumerate many ways in which the local condition may be ameliorated by constitutional treatment. It requires careful attention. As to whether the dentist would be justified in prescribing medicines constitutionally, it depends upon the knowledge the dentist has of the use of the drugs. A physician I know is accustomed to prescribe a compound cathartic pill for toothache. I do not see how that could affect the teeth. Had he used some other constitutional remedy, he might have brought about the desired result. We must know what remedies are to be used and know how to use them; we will then certainly be justified in using them.

*Dr. Gaskill.*—I think that the treatment practised by a good many eminent practitioners is that which I was taught by Professor Litch,—*i.e.*, the use of morphine with a cathartic. I have had results from that treatment which I could not have secured without a cathartic. I think it is the treatment that is prescribed by many practitioners for abortion of an incipient abscess.

*Dr. Schamberg.*—I do not state that cathartics will have no effect on local conditions. I do deprecate, however, the use of cathartics without reference to the nature of the dental condition, whether exposed pulp or apical abscess. The physician referred to was satisfied that giving a cathartic would bring relief in dental diseases generally. The same reasoning applies to purely local treatment. We do not take sufficiently into consideration the primary cause of the dental disease.

*Dr. Luckie.*—Our treatment is principally local, but there are cases where we cannot give relief by local remedies or by mechanical means. These are the cases in which systemic treatment is necessary. We all acknowledge that it is our right and privilege to prescribe it, and I believe that the medical profession grants that we have such right. Should the dentist, however, be so unfortunate as to have a death occur after he has prescribed a systemic remedy, who would give the certificate of death? Can the dentist do it?

*Dr. Truman.*—The coroner in case of accidental death, whether at the hands of a dentist or a physician.

*Dr. Roberts.*—When my patients unexpectedly require a counter-irritant I instruct them to mix a little ginger, red pepper, and mustard and sprinkle a little of this on the fleshy part of a raisin, and then to roast the raisin. This is an easy method of making a capital capsicum plaster in case of emergency. It acts like a charm generally,—sometimes it will not,—but it is more effective than the roasted raisin by itself.

*Dr. Greenbaum.*—I have to express my thanks for the manner in which the paper has been received. In writing it I thought it would be an effrontery to introduce the theories of others. I therefore presented to you my own experiences and results obtained. I recall a number of other points, however, which I did not incorporate in my paper, but these have been thoroughly ventilated by Drs. Guilford and Truman. When we apply anything that is heating to a part, for a short time, it has a stimulating effect; if continued long enough, it results in absolute depression of the part and relaxation, and to produce this pepper-bags are used. We apply the term counter-irritation because we have no better. It draws blood to the part, but after being allowed to remain a long time causes relaxation. We use capsicum because it has a more stimulating effect than the poultice; the heated fig or raisin acts in the same manner, and the relaxation simply enables the pus to work its way through the tissues.

When I have a case of swelling resulting from the formation of abscess, I have no better treatment than the saline cathartic. I have never used a compound cathartic pill. It may be effective by producing general depression, but the saline is the only remedy I know of for reducing a swelling after an abscess has opened.

As to the medico-legal question, the thing has never been tested in a court of law, but most legal authorities claim that the dentist has a right to use anything or any treatment that will aid in relieving a dental condition, and the opinion has been expressed by many learned men that a dentist who has used a remedy in accordance with accepted theories would be exonerated before a court of law in case of accident.

OTTO E. INGLIS,
*Editor Academy of Stomatology.*

